# Spatial and life history variation in a trait-based species vulnerability and impact model

**DOI:** 10.1371/journal.pone.0305950

**Published:** 2024-06-21

**Authors:** Aharon G. Fleury, Casey C. O’Hara, Nathalie Butt, Jaime Restrepo, Benjamin S. Halpern, Carissa J. Klein, Caitlin D. Kuempel, Kaitlyn M. Gaynor, Lily K. Bentley, Anthony J. Richardson, Daniel C. Dunn

**Affiliations:** 1 Centre for Biodiversity and Conservation Science, The School of the Environment, The University of Queensland, St. Lucia, Queensland, Australia; 2 Bren School of Environmental Science and Management, University of California–Santa Barbara, Santa Barbara, California, United States of America; 3 National Center for Ecological Analysis and Synthesis, University of California–Santa Barbara, Santa Barbara, California, United States of America; 4 Australian Rivers Institute, Coastal and Marine Research Centre, Griffith University, Nathan, Queensland, Australia; 5 Departments of Zoology and Botany, University of British Columbia, Vancouver, British Columbia, Canada; 6 CSIRO Environment, Queensland Biosciences Precinct, St Lucia, Queensland, Australia; The University of Auckland - City Campus: University of Auckland, NEW ZEALAND

## Abstract

Anthropogenic pressures threaten biodiversity, necessitating conservation actions founded on robust ecological models. However, prevailing models inadequately capture the spatiotemporal variation in environmental pressures faced by species with high mobility or complex life histories, as data are often aggregated across species’ life histories or spatial distributions. We highlight the limitations of static models for dynamic species and incorporate life history variation and spatial distributions for species and stressors into a trait-based vulnerability and impact model. We use green sea turtles in the Greater Caribbean Region to demonstrate how vulnerability and anthropogenic impact for a dynamic species change across four life stages. By incorporating life stages into a trait-based vulnerability model, we observed life stage-specific vulnerabilities that were otherwise unnoticed when using an aggregated trait value set. Early life stages were more vulnerable to some stressors, such as inorganic pollution or marine heat waves, and less vulnerable to others, such as bycatch. Incorporating spatial distributions of stressors and life stages revealed impacts differ for each life stage across spatial areas, emphasizing the importance of stage-specific conservation measures. Our approach showcases the importance of incorporating dynamic processes into ecological models and will enable better and more targeted conservation actions for species with complex life histories and high mobility.

## Introduction

Regional and global anthropogenic stressors are increasingly disrupting natural processes, resulting in declines in biodiversity across terrestrial, freshwater, and oceanic environments [1–3]. In response, conservation aims to protect biodiversity and mitigate impacts [4]. These efforts are often informed by ecological models, which may provide information such as species’ vulnerability to a stressor or impacts of stressors on a species. However, these models are typically constrained to inputs that are static in space and time [5]. Nature comprises dynamic processes such as species movement, species life histories, and varying stressor intensities. The neglect of these dynamic processes in ecological models is concerning because many species with these characteristics are especially vulnerable to anthropogenic stressors [6–11]. Ecological models that incorporate spatiotemporal variation in species and stressors could enhance the effectiveness of conservation actions for dynamic species by tailoring interventions across space and time [12–14].

Trait-based ecological models are commonly used to evaluate the adaptability and sensitivity of a species to a stressor (i.e., vulnerability) and the potential effects of anthropogenic stressors on a species (i.e., impact). These models have some advantages over other ecological models, as they can reduce data constraints by relating complex biological processes to intrinsic factors (traits) of a species [15,16]. Trait-based models have been used for several types of regional assessments, including to evaluate toxicological impacts from petrochemical spills [17], to manage fisheries in response to climate change [18], and to assess the vulnerability of endemic species to invasive predators [19]. Trait-based models have also been used to estimate vulnerability of a species to multiple stressors on a global scale [20,21]. Butt et al. [20] applied a trait-based framework to estimate the vulnerability of >44,000 marine vertebrate and invertebrate species to 22 anthropogenic stressors, using a total of 42 traits from five categories (movement, reproduction, specialization, spatial scale, and biophysical traits). O’Hara et al. [22] extended this framework by incorporating the spatial distribution of species and stressor intensities to calculate the impact of 13 stressors on 21,267 marine species.

Similar to other static ecological models, trait-based models primarily incorporate trait values that represent an aggregate measure of the trait across a species’ life history, instead of delineating trait values for individual life stages, which could result in imprecise predictions [23]. For instance, carry-over effects from a stressor at one life stage may result in higher vulnerability at a subsequent stage, which is not captured in an aggregated trait value set [24–27]. One study found even minor differences in early life stage habitat conditions, such as coarseness of sedimentation in nursery areas, can result in reduced metabolic rate and size of a species later in life, thereby altering a species vulnerability to stressors in later life stages [28]. Thus, identifying stressors that threaten individual life stages, instead of a species as a whole, could lead to more effective conservation actions [29,30]. Modelling ecosystem-scale impacts for dynamic species is further complicated by spatiotemporal variation in exposures to stressors as species move between key habitat areas, and the stressors themselves may also be dynamic and synergistic [9,31–34]. Creating a spatially-explicit model by coupling species ranges with spatial stressor distributions provides conservation managers with explicit geographic information on where species are most intensely impacted by stressors and facilitates the development of effective conservation actions [22,35,36].

Here we demonstrate the importance of incorporating life history and spatial variation in a species’ distribution in a trait-based vulnerability and impact model using the Butt et al. [20] framework to evaluate vulnerability and impacts from stressors for a part of the metapopulation of the green sea turtle (*Chelonia mydas*) in the Greater Caribbean Region [37,38]. The green sea turtle is a migratory species that undergoes ontogenetic niche shifts and faces multiple stressors along its migratory routes, making it an ideal species to demonstrate implementation of spatiotemporal dynamics in this framework. We incorporate life stages into the Butt et al. [20] framework to observe stressor-specific vulnerability for individual green sea turtle life stages. We further examine how impacts to the green sea turtle shift over space and time by incorporating spatial distributions and stressor magnitudes for each life stage with vulnerability estimates. We explicitly formulated this study to investigate within-species variation spatially and by age class. Understanding the effects of each of these dynamic processes on vulnerability and impact provides insight into how we can improve conservation actions for dynamic species.

## Methods

**Fig 1:**
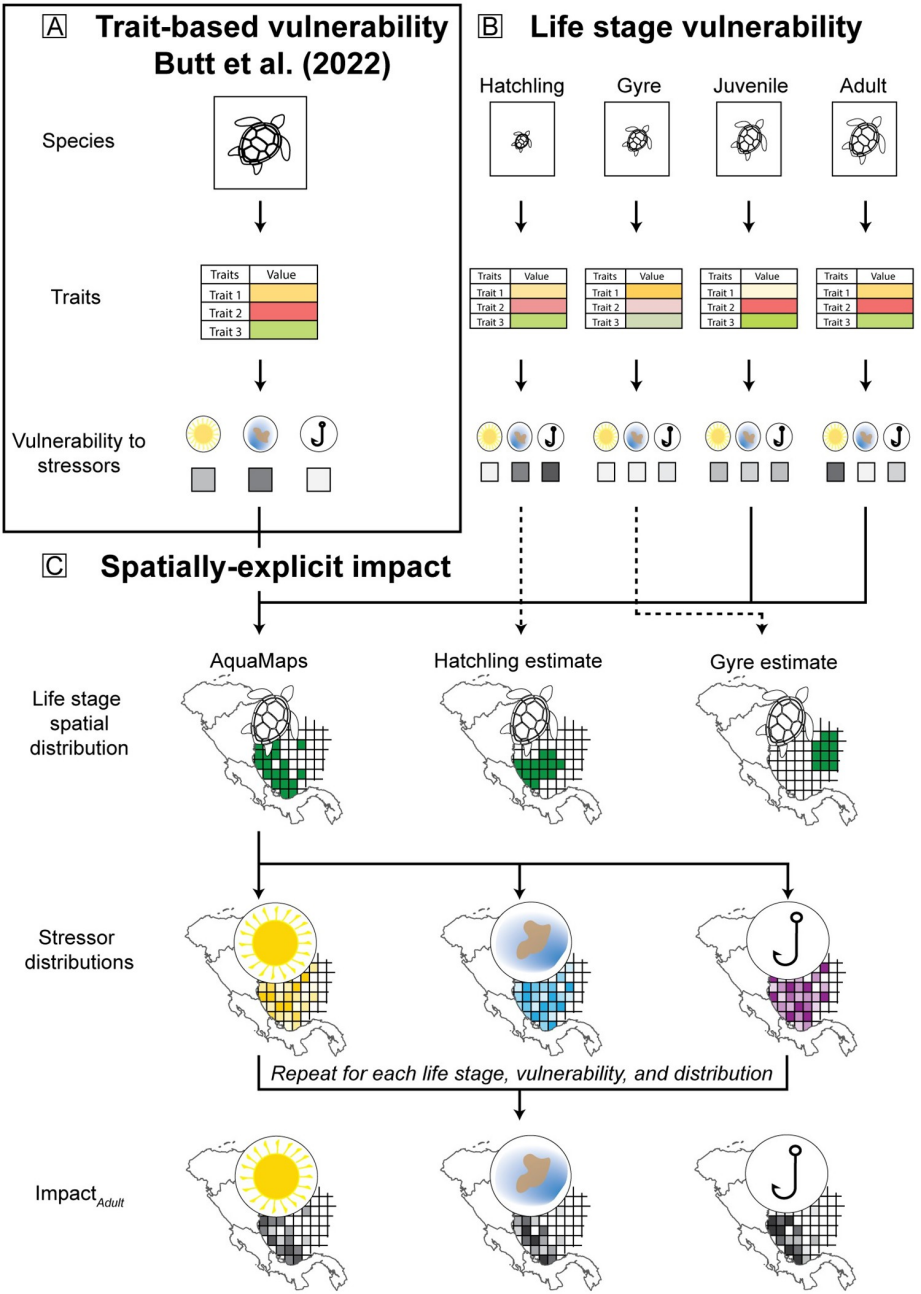
A conceptual diagram of the trait-based vulnerability and impact approach. (A) The Butt et al. (2022) trait-based vulnerability model with (B) the incorporation of life stage variability into the model, and (C) the inclusion of life stage and stressor spatial distributions to calculate spatially-explicit impact. For each trait, each life stage is assessed a trait value represented by the different colour saturations. Vulnerability is calculated for each stressor (e.g., sea surface temperature, pollution, and bycatch) resulting in different vulnerability estimates for each stressor (represented by the grey boxes in (A) and (B)). Life stage and stressor spatial distributions are then incorporated with the vulnerability estimates to calculate impact for each life stage and stressor (represented in (C)). (Turtle icon from stock.adobe.com; Maps made with Natural Earth).

In our approach (summarized in Fig 1), we first evaluated how trait-based vulnerability changes during ontogenetic shifts throughout the life history of the Greater Caribbean green sea turtle metapopulation by dividing its life history into four individual life stages and assigning trait values from the Butt et al. [20] framework for each life stage. We then combined vulnerability estimates with species and stressor intensity distributions to estimate the impact of each stressor on each life stage.

### Green sea turtle life history

The green sea turtle has a global distribution throughout tropical and subtropical waters, spending most of its time in shallow coastal waters [39,40]. As for most sea turtles, green sea turtles are highly migratory and use a wide range of broadly separated localities and habitats during their lifetime [41,42]. As hatchlings, they move from natal beaches to the open pelagic waters, swimming actively offshore until reaching driftline habitat floating in open waters [43]. They then drift, carried by oceanic currents, into ocean gyres, where they reside for several years until they settle into neritic habitats in tropical and temperate zones [44]. Upon reaching adulthood, some turtles move into established foraging habitats in seagrass meadows, although these habitats may also occasionally host younger turtles [45]. Once green sea turtles attain sexual maturity, they embark on seasonal breeding migrations every few years, moving from foraging grounds to courtship and mating areas located offshore from the nesting beach [41,46].

Based on the green sea turtle’s life history, we evaluated its trait-based vulnerability for the metapopulation of the Greater Caribbean region using four life stages: ‘hatchling’, ‘gyre’, ‘juvenile’, and ‘adult’. The first stage, ‘hatchling’, represents the turtle hatchling moving from the nesting beach to the gyre. The second stage, ‘gyre’, represents the period in which the turtle resides in the gyre of the Caribbean and Sargasso Sea, otherwise known as the ‘lost years.’ The third stage, ‘juvenile’, represents the nomadic years of a turtle after it leaves the gyre to forage along coastlines prior to the first breeding season and establishment of a permanent foraging area. The fourth stage, ‘adult’, represents the turtle post-first breeding, where it has established a specific foraging range. We also re-calculated the vulnerability estimate using the trait values for the green sea turtle from the Butt et al. [20] framework to compare differences in the approaches. From the original 42 traits in the Butt et al. [20] framework, we identified seven traits with values that could be applied to specific life stages for the green sea turtle (Table 1). The remaining 35 trait values were fixed across all stages. To maintain standardisation with the original framework we did not change or add traits or trait values from Butt et al. [20]. As a result, this application of the framework may not use traits or trait values that should be included in a more detailed, management-relevant model of impact on green sea turtles and should simply be taken as a demonstration of how the Butt et al. [20] framework can be expanded to incorporate spatiotemporal dynamism across life history stages. For example, a full trait-based analysis of impacts on green sea turtles would incorporate chemoreception navigational capabilities as a trait [47,48]. For the seven traits with ontogenetic shifts, we identified trait values for each life stage from our expert knowledge and the available literature [49–51].

**Table 1:** Green sea turtle traits modified from the original Butt et al. **[20] framework.** Shown here are the seven traits from the Butt et al. [20] framework with trait values that can be subdivided for individual species’ life stages. The full 42 traits and associated trait values for each life stage of the green sea turtle are available in the S1 Table.

Trait	Trait values
Adult mobility	Sessile, nearly sessile/sedentary, passive, vertical migrator, mobile resident, horizontal migrator, nomadic
Thermal sensitivity to heat waves	Yes, no, NA
Dependent habitats	Surface water, beach, continental shelf, coral reef, seagrass
Extreme diet specialization	Yes, no, NA
Zone	Intertidal, neritic, oceanic, pelagic, demersal, benthic
Body size	<0.4 mm, 0.5–49 mm, 50–999 mm, >1000 mm
Navigation requirements	Sound, light, magnetic

### Trait-based vulnerability model

We extended the trait-based vulnerability model from Butt et al. [20] and O’Hara et al. [22] by including physiological and life history traits to estimate life stage sensitivity and adaptive capacity to a stressor. Vulnerability (*V*_*ij*_), defined as the relative effect of stressor *j* on the health of life stage *i*, was estimated using the Butt et al. [20] framework vulnerability calculation.


$$V_{ij}=\frac{\frac{S_{ij}}{S_{ij}^{max}}}{1+\frac{A_{ij}}{A_{ij}^{max}}+\frac{G_{i}}{G_{i}^{max}}}\times E_{ij}$$ (1)

*V*_*ij*_ is based on the presence of certain traits that are likely to increase the physiological sensitivity *S*_*ij*_ of life stage *i* to stressor *j*, other traits that affect the adaptive capacity *A*_*ij*_ of life stage *i* to stressor *j*, and life history and population-level traits that affect the general adaptive capacity *G*_*i*_ of life stage *i* to adapt to or recover from disturbances in general. To ensure that *V*_*ij*_∈[0,1], i.e., *V*_*ij*_ takes values between 0 and 1 inclusive, variables *S*_*ij*_, *A*_*ij*_ and *G*_*i*_ are scaled relative to their maximum values across all life stages, i.e., $S_{ij}^{max},A_{ij}^{max}$, and $G_{i}^{max}$. An additional exposure modifier was included to account for the possibility of exposure *E*_*ij*_∈{0,1} of life stage *i* to stressor *j*. For example, a life stage in the intertidal zone will not be exposed to ship strikes so *E*_*ij*_ = 0. All quantities on the right-hand side of the equation are unitless, ensuring *V*_*ij*_ is also unitless. Uncertainty in the vulnerability estimates for each life stage cannot be estimated as only a singular value is calculated for each life stage. This is a result of the structure of the trait-based vulnerability framework and the categorical values for traits.

### Life history stage and stressor spatial distribution

**Fig 2:**
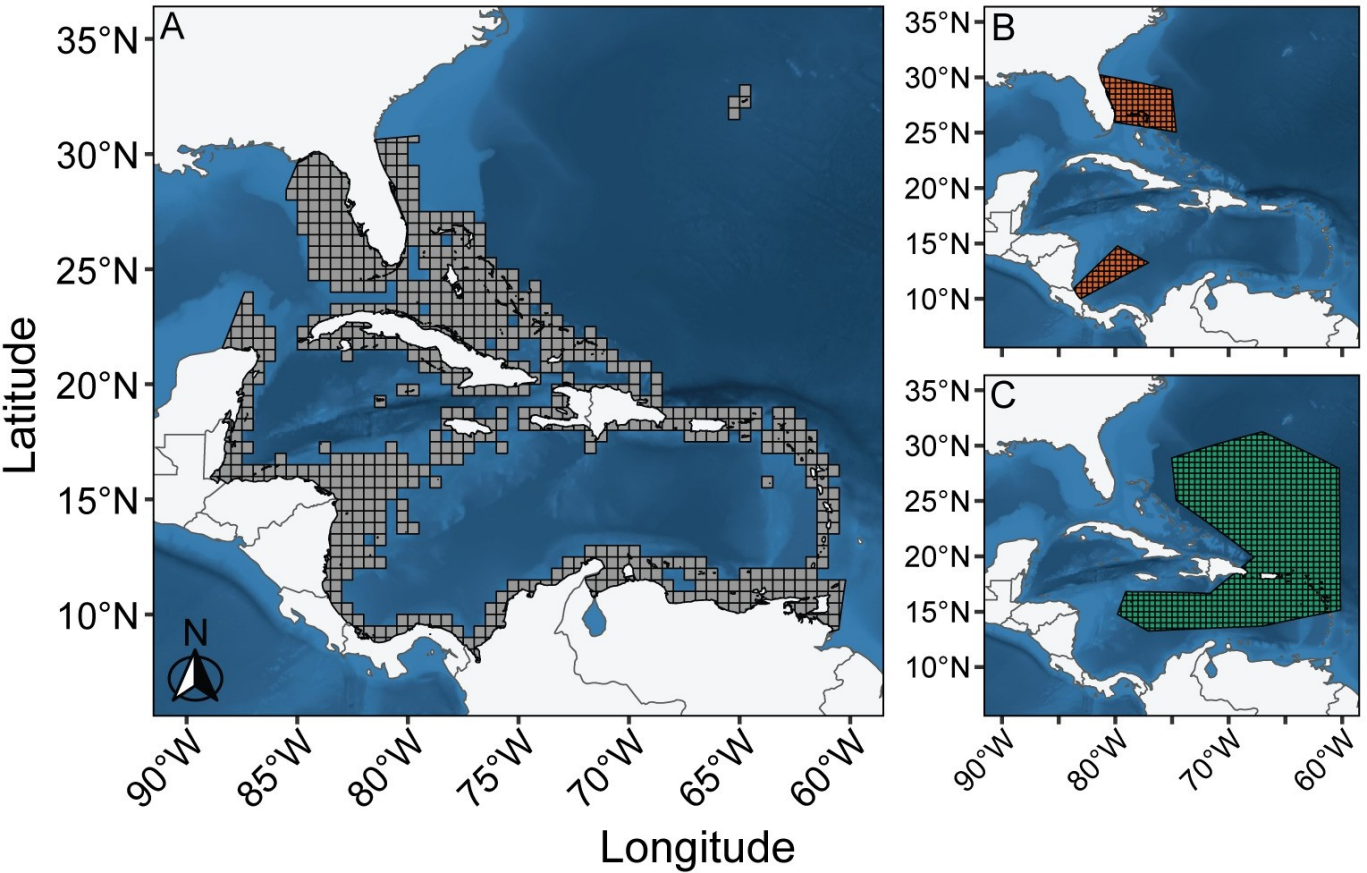
Comparison of spatial distributions for green sea turtle life stages in the Greater Caribbean Region. (A) An AquaMaps distribution for green sea turtles bounded to a specified area of the Greater Caribbean Region. (B) An example spatial distribution for a hatchling life stage from two major nesting beaches. (C) An example spatial distribution for a gyre life stage. Each grid cell is 0.5° x 0.5°. Note that the polygons for (B) and (C) are merely estimates and may not represent actual distributions for these life stages from these specific beaches. (Maps made with Natural Earth).

To align with previous studies, we acquired species distribution data for the green sea turtle from AquaMaps and bounded it to a specified area of the Greater Caribbean Region [52] (Fig 2A). AquaMaps estimates the probability of occurrence of a species in 0.5° x 0.5° grid cells based on environmental preferences with respect to depth, sea surface temperature, salinity, proportional ice cover, and primary productivity. One limitation of Aquamaps is that it uses a single distribution to describe the entire life history of a species and is typically representative of adult movement patterns. We estimated additional turtle life stage distributions for ‘hatchling’ and ‘gyre’ stages based on the available literature (Fig 2B and 2C). Some studies have estimated movement patterns for early life stages within the Greater Caribbean Region using models or telemetry data, but movement data for neonate turtles remains sparse and no consensus on their distribution exists [53]. Model movement estimates generally assume that early life stages drift with oceanographic currents [43,54,55], however, this is contradicted by tracking studies that show turtles actively swimming against the prevailing current [56–60]. Thus, our ‘hatchling’ and ‘gyre’ polygons are just one potential estimate of their distribution. However, the exact distribution is inconsequential to the results of this study, as the polygons are primarily used to show that the Butt et al. [20] framework and species’ distribution databases (e.g., AquaMaps) neglect early life stage spatial distributions, which undermines estimates of impacts. The estimated hatchling distribution comprises two polygons that stretch from two major nesting beach regions, one along the eastern coast of Florida and the other along the Caribbean coastline of Costa Rica [37], out towards either the Sargasso Sea or Caribbean gyres, respectively. The estimated gyre distribution is a single polygon that encompasses both the Sargasso Sea gyre and the Caribbean gyre. We divided the estimated polygons for the hatchling and gyre distributions into 0.5° x 0.5° grid cells to be consistent with the AquaMaps distribution grid. These estimated distributions illustrate the potential effect of spatial variation for a life stage with different spatial distributions but should be revised for green sea turtle specific studies.

We used the following rasters of anthropogenic stressors at 10 km resolution, developed by O’Hara et al. [22], to assess 9 of the 22 stressors used in the vulnerability calculations: bycatch, ocean acidification, sea level rise, marine heat waves, ship movement, ultraviolet radiation, nutrient pollution, light pollution, and habitat degradation (S1 Appendix). Stressor data originally captured information at a finer resolution than the species distribution polygons. As a result, the stressor data were area-weighted to the polygons in the AquaMaps distribution as well as the estimated ‘gyre’ and ‘hatchling’ distribution polygons. All spatial data were transformed into a Lambert azimuthal equal-area projection and bounded to the Greater Caribbean region.

### Spatially explicit impact of stressors

The impact *I*_*ijn*_ on life stage *i* to stressor *j* in cell *n* was calculated as the product of the trait-based vulnerability estimate *V*_*ij*_ for life stage *i* to stressor *j*, the stressor intensity *T*_*jn*_ for stressor *j* in cell *n*, and life stage presence *P*_*in*_ for life stage *i* in cell *n*.


$$I_{ijn}=V_{ij}T_{jn}P_{in}$$ (2)

Life stage presence for cell *n* is based on AquaMaps probability of occurrence data or an assumed probability of 1 for the estimated gyre and hatchling distributions. All quantities on the right-hand side of the equation are unitless, ensuring *I*_*ijn*_ is also.

The mean impact *I*_*ij*_ on life stage *i* to stressor *j* across all cells *n*, given the total number of cells *N*_*i*_, was then calculated to compare stressor impacts within and across life stages. All quantities on the right-hand side of the equation are unitless, ensuring *I*_*ij*_ is also.


$$I_{ij}=\frac{1}{N_{i}}\sum_{n=1}^{N_{i}}I_{ijn}$$ (3)

### Statistical software and code

Analyses were performed using the R statistical software [61], version 4.3.1, using the following packages: tidyverse [62], rnaturalearth[63], sf [64,65], and terra [66]. We modified the trait-based vulnerability model code from Butt et al. [20] and O’Hara et al. [22] for our study. Detailed methods, code, intermediate data, and output data for this study may be found at https://github.com/aharon-fleury/vuln_framework_case_studies.

## Results

### Vulnerability to stressors varied across life stages

**Fig 3:**
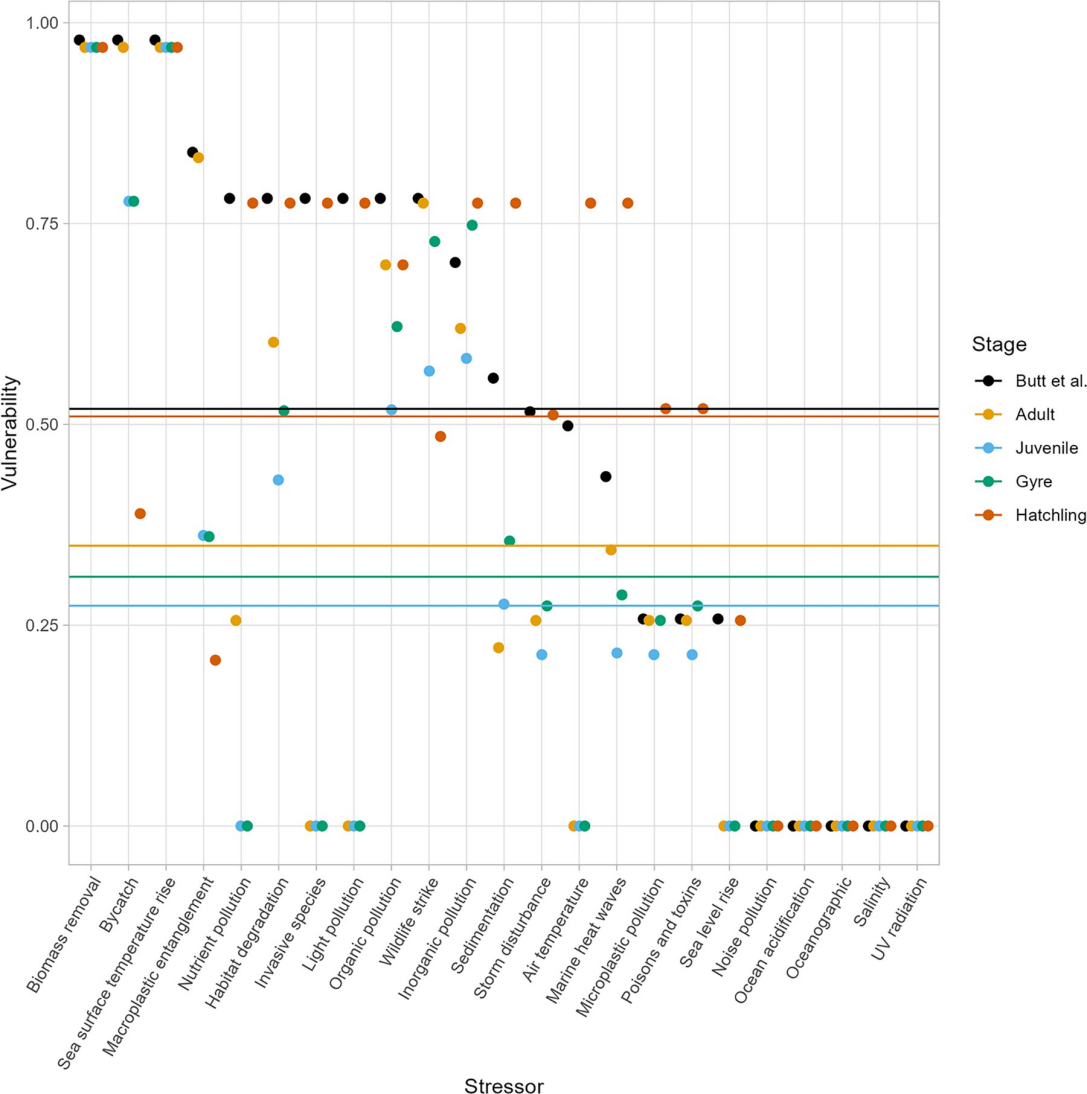
Green sea turtle trait-based vulnerability to 23 individual stressors. Vulnerability shown without consideration of life-history stages (Butt et al.) and with consideration of four life stages using different colours for each stage. The mean vulnerability to all stressors for each life stage is represented by the horizontal lines. Each point represents a single estimate of trait-based vulnerability for each life stage and stressor.

When compared to the Butt et al. estimate, vulnerability to each stressor varied for each green sea turtle life stage because of the stage-specific trait value changes for 7 of the 42 traits (Fig 3). On average, the four separate life stages resulted in a lower vulnerability across all stressors when compared to the Butt et al. estimate. Hatchlings were more vulnerable to stressors compared to the other life stages, but almost equivalent to the Butt et al. estimate. Vulnerability decreased from the hatchling through the gyre to the juvenile stages, and then increased to the adult stage. Only the hatchling and gyre stages had vulnerabilities to individual stressors greater than the Butt et al. estimate. The hatchling had vulnerabilities greater than the Butt et al. estimate for 6 of the 22 stressors: inorganic pollution, sedimentation, air temperature, marine heat waves, microplastic pollution, and poisons and toxins. The gyre had vulnerabilities greater than the Butt et al. estimate for 2 of the 22 stressors: inorganic pollution and poisons and toxins. The juvenile vulnerabilities were less than or equivalent to the adult stage for all stressors except for sedimentation. The adult had vulnerabilities less than the Butt et al. estimate for 11 of the 22 stressors: nutrient pollution, habitat degradation, invasive species, light pollution, organic pollution, inorganic pollution, sedimentation, storm disturbance, air temperature, marine heat waves, and sea level rise.

### Impact of stressors varied spatially

**Fig 4:**
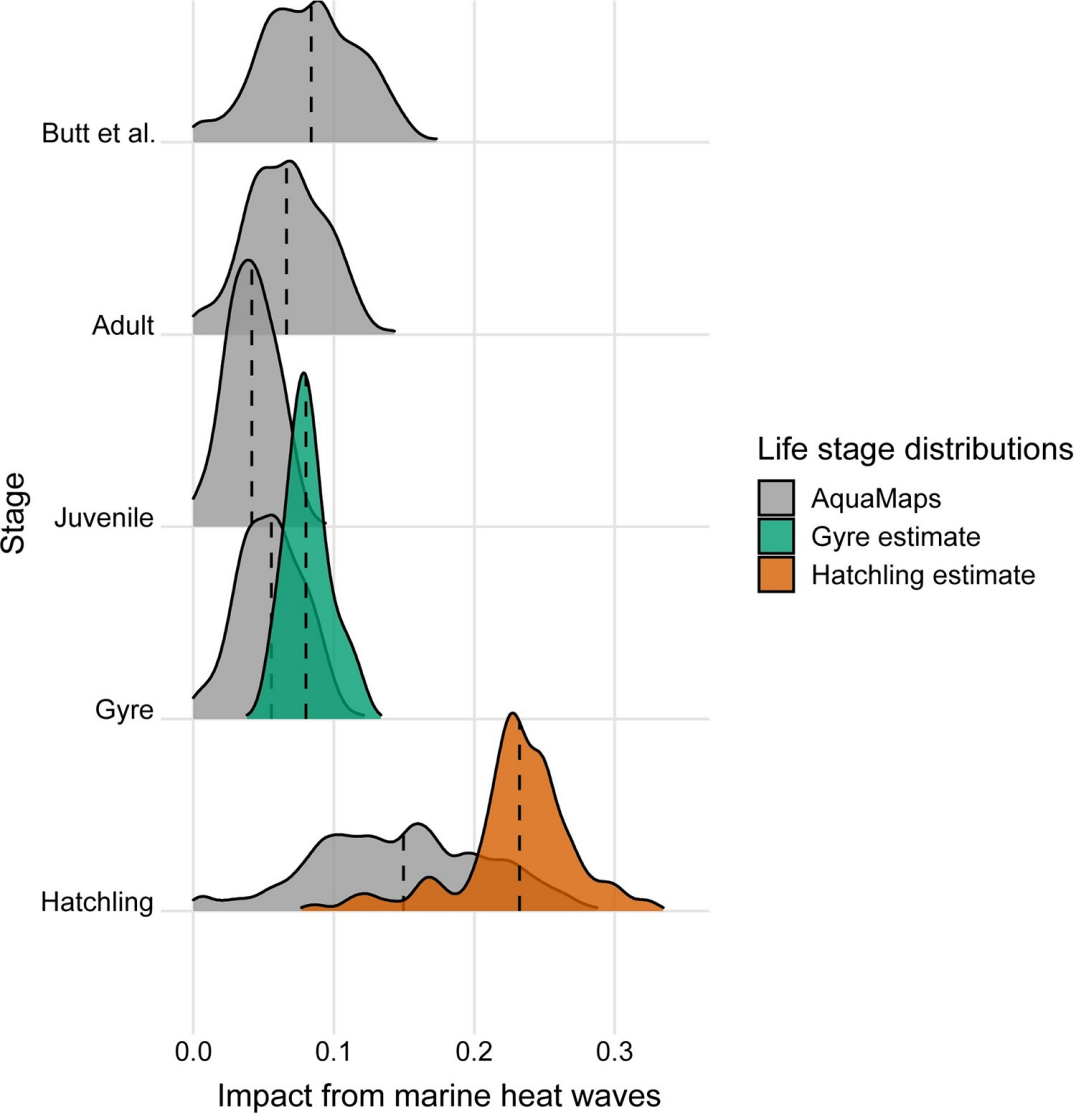
Kernel density estimates of the impact on the green sea turtle of marine heat waves. The kernel density estimates reflect the distribution of grid cell impact estimates for marine heat waves across the spatial distribution for each life stage. Impacts from the stressor were calculated for three spatial distributions: AquaMaps (grey), the gyre polygon estimate (green), and the hatchling polygon estimate (orange). The variability in each life stage compared to the Butt et al. stage exemplifies the effect of stressor spatial distributions for individual life stages of a species. Comparing within life stages for gyre and hatchling distributions shows the effect of life stage distributions compared to adult-focused species distributions such as AquaMaps.

Applying spatial stressor intensities and life-stage distributions to 9 of the 22 stressors from the vulnerability model, we found that impacts of stressors on green sea turtles in the Greater Caribbean region varied spatially for each life stage and the Butt et al. estimate. Overall, most stressors had higher impacts nearshore and impacts declined substantially offshore due to the nature of the stressor. For most stressors, there was limited variability across life stages (S2 Appendix). This was primarily due to the structure of the framework and the lack of ability to provide more continuous distributions of trait values. Impacts from ocean acidification and ultraviolet radiation were zero as the vulnerabilities for each life stage were zero. When evaluating the impact among stages, large variation in impacts were identified for individual stressors, including marine heat waves and bycatch. Impact of marine heat waves on hatchling and gyre life stages increased when using the estimated life-stage distributions compared to the AquaMaps species distribution (Fig 4). The opposite occurred for bycatch, with impact decreasing relative to the AquaMaps distribution for both hatchling and gyre life stages when using their estimated life-stage distributions.

## Discussions

Species face varying intensities of anthropogenic stressors across space and time [8,9], yet these dynamic interactions between species and threats are not captured by static models [9,13,14]. As many species move across ecosystems and jurisdictions throughout their life history, it is imperative to capture those dynamics in models to maximize the chance for effective conservation outcomes. Using green sea turtles as our demonstration and separating the traits in the framework into multiple life stages, we identified vulnerability to specific stressors that were not observed when using aggregated life history trait values (i.e., the Butt et al. estimate). Further, incorporating spatial components into each life stage of the green sea turtle provided insight into key stressor impact areas for individual life stages that would go unnoticed when focusing on an entire species distribution. Thus, delineating vulnerability and impact of a dynamic species through space and time provides a more effective assessment of the stressor impacts and allows for more targeted conservation measures.

Our model results show that the Butt et al. estimate captured the general vulnerability of the green sea turtle across multiple stressors, however, it did not adequately capture life stage-specific vulnerabilities. While overall vulnerability scores were similar, by dividing the green sea turtle life history into four stages, we identified higher vulnerabilities to specific stressors (e.g., inorganic pollution and marine heat waves) during earlier rather than later life stages. Mitigation of stressor impacts in early life stages is critical to avoid potentially harmful carry-over effects, which occur when an individual’s previous environmental experiences affects their current performance [28,67]. Carry-over effects may have negative effects, such as long-term sublethal effects that reduce resilience to future stressors. However, carry-over effects may also have positive effects, whereby individuals become more resilient to future stressors resulting in enhanced survival and reproductive output [28,67]. Carry-over effects may occur from as early as the egg stage in turtles [28]. For example, warming incubation temperatures of sea turtle eggs can impact individual behaviour, physiology, morphology, and performance, and may have long-term impacts on sex ratios resulting in reduced species or population fitness [68–70]. Although our model does not quantitatively consider carry-over effects across life stages, it does provide valuable insight into which life stages are vulnerable to specific stressors.

By combining distributions of life stages and stressors, we observed changes in the estimated impact of threats across regions and throughout the life cycle. In addition, our model shows how spatial distributions from aggregate databases may neglect information for specific life stages resulting in inaccurate assessments of species’ vulnerability to and impact from stressors. These spatial and temporal analyses can be used to identify high and low impact areas that exemplify different interactions of stressor intensities and vulnerability [22]. Conservation management may benefit from the use of explicit spatial information to plan potential protected areas based on impact to specific life stages. However, this approach could be limited on a local scale, as the spatial resolution of major anthropogenic stressors, such as those related to climate change, and species distribution data, are typically too coarse. Therefore, on a local scale it is necessary to collect finer resolution data on anthropogenic stressors and species distributions to appropriately plan protected areas.

Spatiotemporally explicit trait-based models can provide a more realistic picture of the vulnerability of dynamic species to stressors across jurisdictions than those that do not consider different life history stages. Conservation planning without accounting for spatiotemporal dynamism may lead to ineffective management strategies [9,71]. For species-specific management, considering multiple life stages allows for strategies to be developed to mitigate stressor effects on individual life stages, which may otherwise be ignored at the species level. With the addition of life stage distributions and stressor intensity distributions, management strategies may identify high and low impact areas to mitigate impact effectively for specific life stages. The need to include spatiotemporal variation into models will become even more acute as efforts shift toward dynamic spatial management [36,72]. Management strategies could apply multiple life stage time-of-use closures across specific zones as a more cost-effective approach instead of a single larger blanket closure for the species. Explicit inclusion of time and space data into models may be more costly initially in terms of data collection but will likely lead to a more effective and successful conservation strategy in the long-term.

Although incorporating spatiotemporal information enhances our understanding of species’ vulnerabilities, such models still have limitations. In the current trait-based vulnerability framework, uncertainty around the mean for each life stage cannot be calculated as each vulnerability estimate is a singular value for each life stage due to the categorical nature of the trait values. If the trait values represented a distribution for each trait, then uncertainty for each life stage could be calculated. As with all models, trait-based vulnerability models are limited by available input data on traits, vulnerabilities, species distributions, and stressor distributions. Traits can easily quantify intrinsic factors within each life stage, but intraspecific trait variation is lost when binning into single categorical trait values, which may reduce confidence in vulnerability estimates. In addition, expert subjectivity when categorizing traits and their vulnerability inherently introduces bias, particularly as traits move away from objective information. By subjectively dictating whether to include or exclude a trait, we increase the likelihood of highly variable vulnerability estimates. As a result, it is necessary to identify key trait categories that are sufficiently specific to capture a species’ intrinsic factors but simultaneously broad enough to be used for other species [73,74]. Species distributions, such as those in AquaMaps, are typically aggregated across all life stages or target adult distributions resulting in the loss of stage-specific dynamics that would otherwise be captured if the distributions were delineated by life stage. Stressor distributions are often averaged over time scales larger than certain life stages, thus, the magnitude and spatial extent of stressors need to be refined to appropriate time scales relevant to specific life stages. For example, annual averages may wash out acute events such as the difference between marine heat waves and annual sea-surface temperatures. Overall, the uncertainty in these trait-based vulnerability and impact models may be reduced by addressing these limitations.

Future trait-based vulnerability models could be strengthened by reducing human subjectivity in trait selection and categorization. One method may be to use probabilistic simulations and statistical distributions of quantified traits bounded by minimum and maximum values. Simulations that capture the extremes may then be considered potential iterations of early and late life stages. If trait-based vulnerability models are to be used for conservation purposes then, at a minimum, a standard set of traits that effectively capture species vulnerability needs to be identified so that measures may be consistent among conservation areas [75]. Future models could also incorporate carry-over effects to better understand species vulnerability in later life stages.

Our study used green sea turtles to demonstrate the effects of incorporating spatiotemporal dynamics into a trait-based vulnerability and impact framework. To do so, we maintain as much of the original framework as possible to allow for standardization and comparison of results. This also resulted in a generalized perspective of green sea turtle life history, which did not incorporate all traits or trait values for green sea turtles. More specific information is necessary when assessing risk [76] or management [77] of green sea turtles. Future studies that aim to better understand the vulnerability to and impacts from stressors for green sea turtles could implement a more detailed approach of the species’ life history. Such details could include incorporation of temporal elements into the life stage distributions, the proportion of time a life stage is exposed to a specific stressor, or the proportion of a population exposed to a stressor at a specific time. Traits and trait values that were not considered in this framework due to standardization could also be incorporated. For example, trait values such as navigational capabilities (i.e. chemoreception) may identify further species’ vulnerabilities to and impacts from stressors [47,48]. Finer spatial distributions for each of the four life stages could be used, as well as time spent in each location relative to the presence of the stressor [54,76,77]. Such species-specific details could be considered alongside incorporation of uncertainty and model enhancements for the vulnerability and impact models.

The addition of spatial and temporal information into a trait-based vulnerability model can provide a more accurate estimate of a species vulnerability to anthropogenic stressors, particularly for dynamic species like the green sea turtle used for demonstration here. The incorporation of spatial information provides conservation decision-makers with focused areas of interest for individual life stages instead of across the entirety of a species range. This thereby affords the option for life stage-specific conservation actions that may be more effective than actions over an entire species’ range across all life stages. Applying dynamic trait-based models should support more effective prioritization of conservation actions for dynamic species.

## Supporting information

S1 Table Green sea turtle traits and trait values for each life stage. (PDF)

S1 Appendix Anthropogenic stressor intensity maps. (PDF)

S2 Appendix Boxplots of stressor impacts. (PDF)
