# Supplementary material for: Spatial and life history variation in a trait-based species vulnerability and impact model
Source: PLoS One. 2024 Jun 21;19(6):e0305950. doi: 10.1371/journal.pone.0305950 (PMC11192397; doi:10.1371/journal.pone.0305950)
Supplement: S1 Table — (PDF) [file pone.0305950.s001.pdf]

PLOS One

Spatial and life history variation in a trait-based species vulnerability and impact model

Aharon G. Fleury, Casey C. O'Hara, Nathalie Butt, Jaime Restrepo, Benjamin S. Halpern, Carissa J. Klein, Caitlin D. Kuempel, Kaitlyn M. Gaynor, Lily K. Bentley, Anthony J. Richardson, Daniel C. Dunn

S1 Table

S1 Table. Green sea turtle (*Chelonia mydas*) traits and trait values for four life stages and the original Butt et al. estimate.

**Trait**	**Chelonia mydas (Butt et al.)**	**Chelonia mydas (Adult)**	**Chelonia mydas (Juvenile)**	**Chelonia mydas (Gyre)**	**Chelonia mydas (Hatchling)**
biomass removal sensitivity trait	sensitive	sensitive	sensitive	sensitive	sensitive
adult body mass/body size	>1000mm	>1000mm	50mm-999mm	50mm-999mm	0.5mm-49mm
calcium carbonate structure location	none	none	none	none	none
calcium carbonate structure stages	none	none	none	none	none
communication requirement (sound)	no	no	no	no	no
extreme pressure wave sensitive structures	unknown	unknown	unknown	unknown	unknown
flight	no	no	no	no	no
navigation requirements (light)	yes	NA	NA	NA	yes
navigation requirements (magnetic)	yes	yes	yes	yes	yes
respiration structures	lungs	lungs	lungs	lungs	lungs
if one/few, size	na	na	na	na	na
number of sites	many	many	many	many	many

Trait	Chelonia mydas (Butt et al.)	Chelonia mydas (Adult)	Chelonia mydas (Juvenile)	Chelonia mydas (Gyre)	Chelonia mydas (Hatchling)
sub-population dependence on particular sites	yes	yes	yes	yes	yes
if one/few, size	na	na	na	na	na
number of sites, incl. terrestrial wetlands	does not aggregate	does not aggregate	does not aggregate	does not aggregate	does not aggregate
sub-population dependence on particular sites	no	no	no	no	no
adult mobility	horizontal migrator	nomadic	horizontal migrator;nomadic	mobile resident	horizontal migrator
planktonic larval duration (pld) exposure	not larvae	not larvae	not larvae	not larvae	not larvae
dissolved oxygen	air breathers	air breathers	air breathers	air breathers	air breathers
ph	<7.4	<7.4	<7.4	<7.4	<7.4
salinity	na	na	na	na	na
sensitivity to wave energy (physical forcing)	not sensitive	not sensitive	not sensitive	not sensitive	not sensitive
sst rise sensitivity trait	sensitive	sensitive	sensitive	sensitive	sensitive
thermal - sensitivity to heat spikes/heat waves	no	no	no	no	yes
thermal - sensitivity to ocean warming - max temps tolerated	30c	30c	30c	30c	30c
age to 1st reproduction/generation time	>20yrs	>20yrs	>20yrs	>20yrs	>20yrs
are there sub-populations?	yes	yes	yes	yes	yes
can the sex ratio be altered by temperature?	yes	yes	yes	yes	yes

**Trait**	**Chelonia mydas (Butt et al.)**	**Chelonia mydas (Adult)**	**Chelonia mydas (Juvenile)**	**Chelonia mydas (Gyre)**	**Chelonia mydas (Hatchling)**
fecundity	100-1000	100-1000	100-1000	100-1000	100-1000
feeding larva (post-hatching metamorphosis)	no larva	no larva	no larva	no larva	no larva
global population size	1k-10k;10k-100k	10k-100k	10k-100k	10k-100k	10k-100k
lifetime # reproductive opportunities	51-100	51-100	51-100	51-100	51-100
max age	20-100yrs	20-100yrs	20-100yrs	20-100yrs	20-100yrs
parental investment	egg-layer	egg-layer	egg-layer	egg-layer	egg-layer
post-birth/hatching parental dependence	na	na	na	na	na
reproductive strategy	sexual dioecious	sexual dioecious	sexual dioecious	sexual dioecious	sexual dioecious
depth (min/max)	epipelagic;air	epipelagic	epipelagic	epipelagic	epipelagic;air
eoo (range)	>20000	>20000	>20000	>20000	>20000
zone	neritic;oceanic	neritic	neritic;oceanic	neritic;oceanic	intertidal;neritic;oceanic
across-stage dependent habitats + condition	coral reef; seagrass; surface water; beach; continental shelf	surface water; beach; seagrass	surface water; seagrass; continental shelf	surface water; continental shelf	surface water; beach; continental shelf
air-sea interface	yes	yes	yes	yes	yes
dependent interspecific interactions	no	no	no	no	no
extreme diet specialization	generalist	specialist	generalist	generalist	na
habitat forming	no	no	no	no	no
photosynthetic	no	no	no	no	no
terrestrial and marine life stages	yes	no	no	no	yes

Trait	Chelonia mydas (Butt et al.)	Chelonia mydas (Adult)	Chelonia mydas (Juvenile)	Chelonia mydas (Gyre)	Chelonia mydas (Hatchling)
within-stage dependent habitats + condition	coral reef; seagrass; surface water; beach; continental shelf	surface water; beach; seagrass	surface water; seagrass; continental shelf	surface water; continental shelf	surface water; beach; continental shelf
