# Supplementary material for: Spatial and life history variation in a trait-based species vulnerability and impact model
Source: PLoS One. 2024 Jun 21;19(6):e0305950. doi: 10.1371/journal.pone.0305950 (PMC11192397; doi:10.1371/journal.pone.0305950)
Supplement: S1 Appendix — (PDF) [file pone.0305950.s002.pdf]

PLOS One

Spatial and life history variation in a trait-based species vulnerability and impact model

Aharon G. Fleury, Casey C. O'Hara, Nathalie Butt, Jaime Restrepo, Benjamin S. Halpern, Carissa J. Klein, Caitlin D. Kuempel, Kaitlyn M. Gaynor, Lily K. Bentley, Anthony J. Richardson, Daniel C. Dunn

S1 Appendix

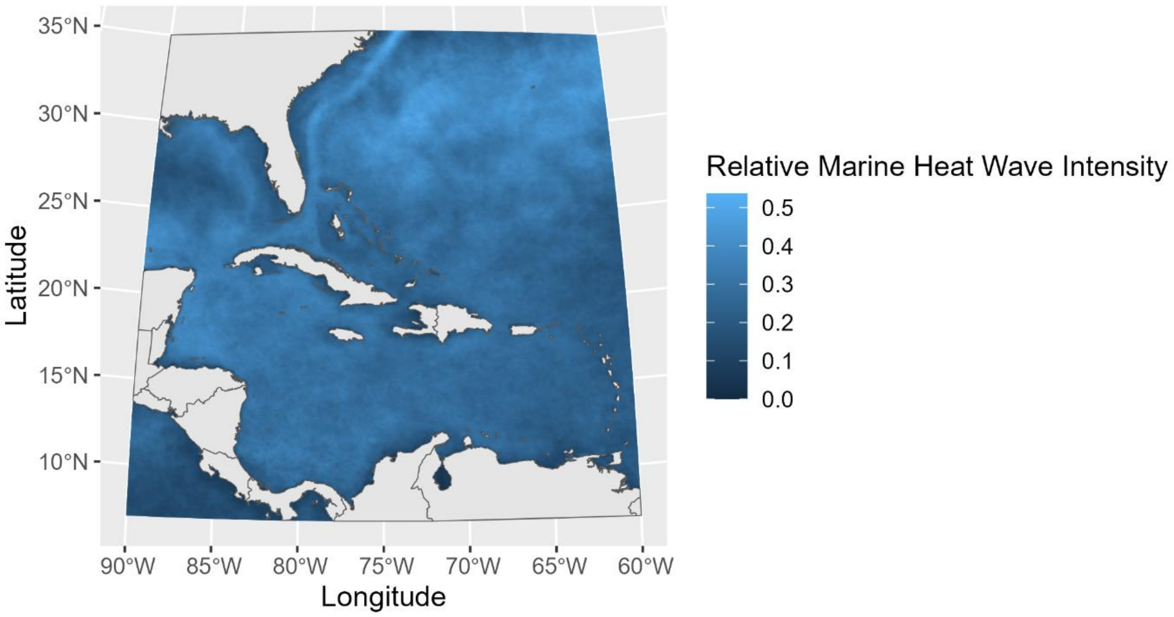

Figure 1. Relative marine heat wave intensity stressor map. Stressor data and methods of collection are available in O'Hara [22]. Dark grey values represent areas where information is not available. Base maps made with Natural Earth.

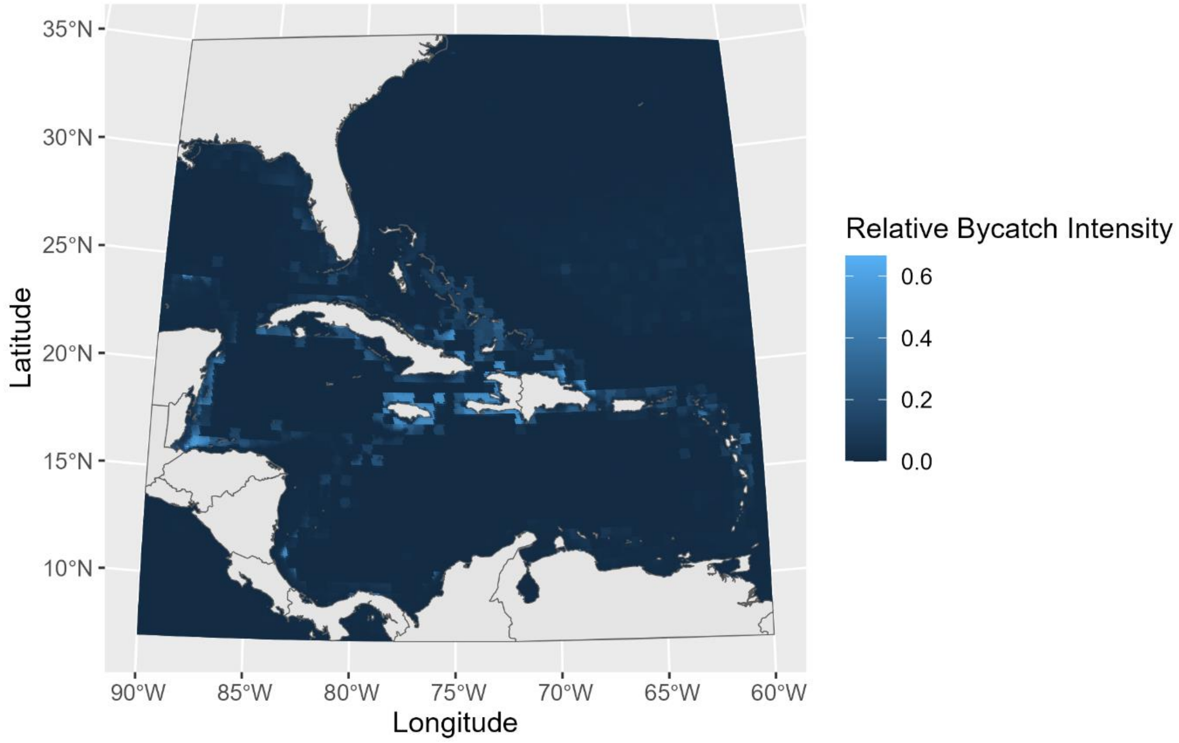

Figure 2. **Relative benthic and pelagic bycatch intensity stressor map.** Stressor data and methods of collection are available in O'Hara [22]. Dark grey values represent areas where information is not available. Base maps made with Natural Earth.

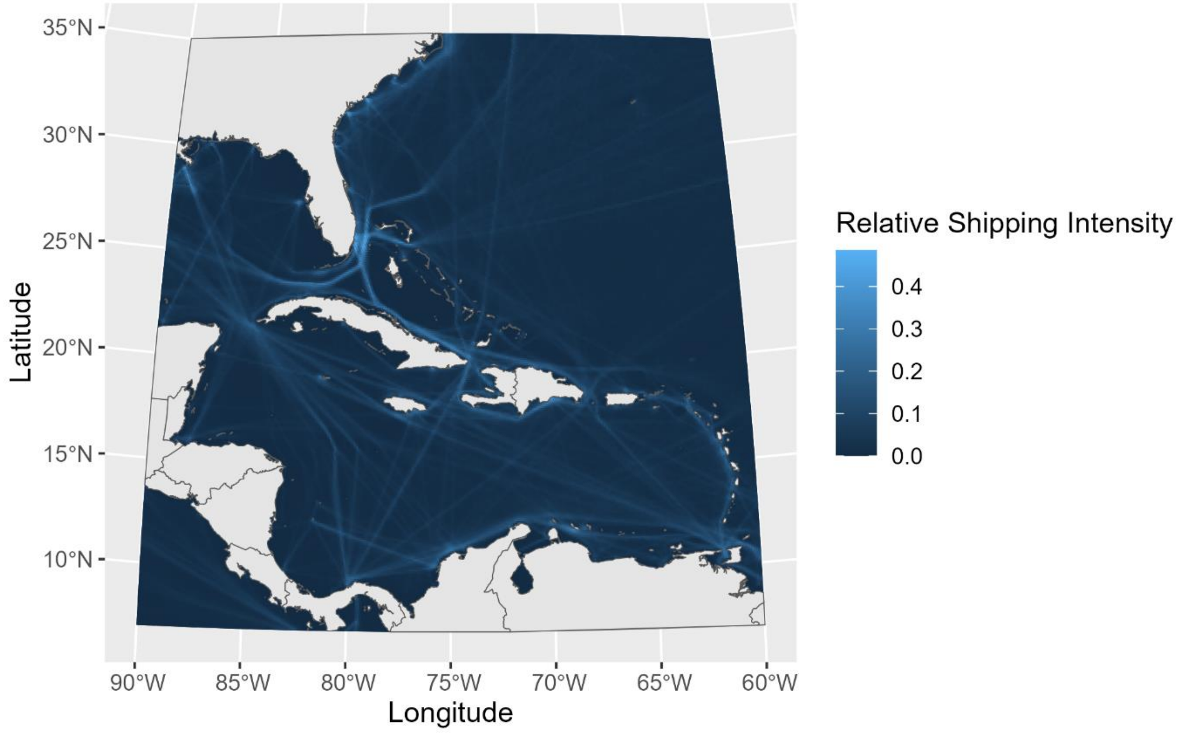

Figure 3. Relative shipping intensity stressor map. Stressor data and methods of collection are available in O'Hara [22]. Dark grey values represent areas where information is not available. Base maps made with Natural Earth.

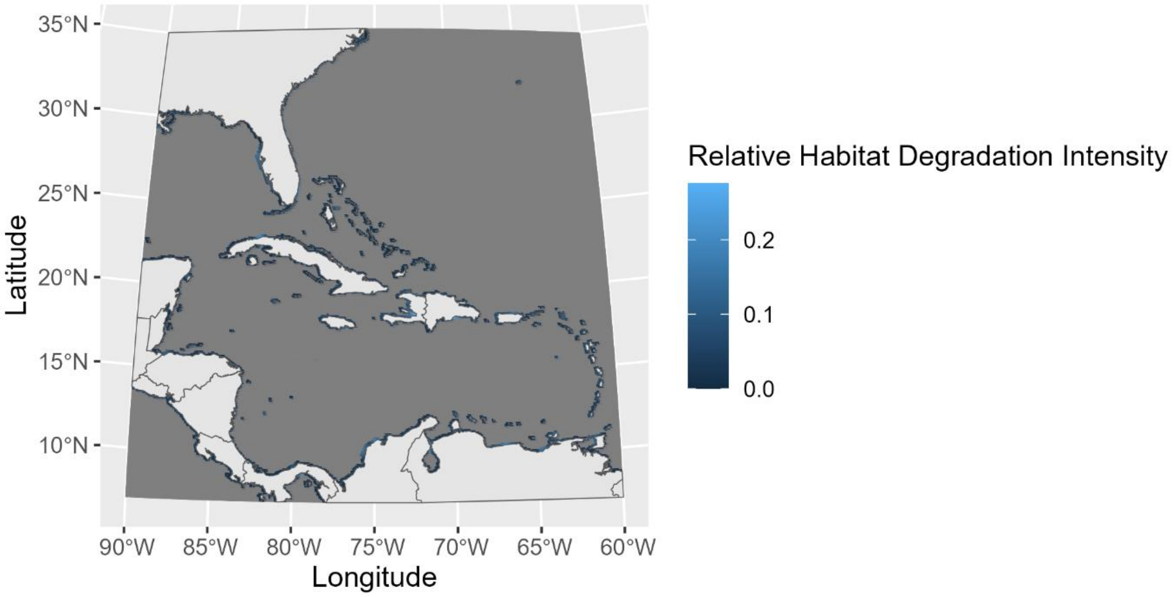

Figure 4. Relative habitat degradation intensity stressor map. Stressor data and methods of collection are available in O'Hara [22]. Dark grey values represent areas where information is not available. Base maps made with Natural Earth.

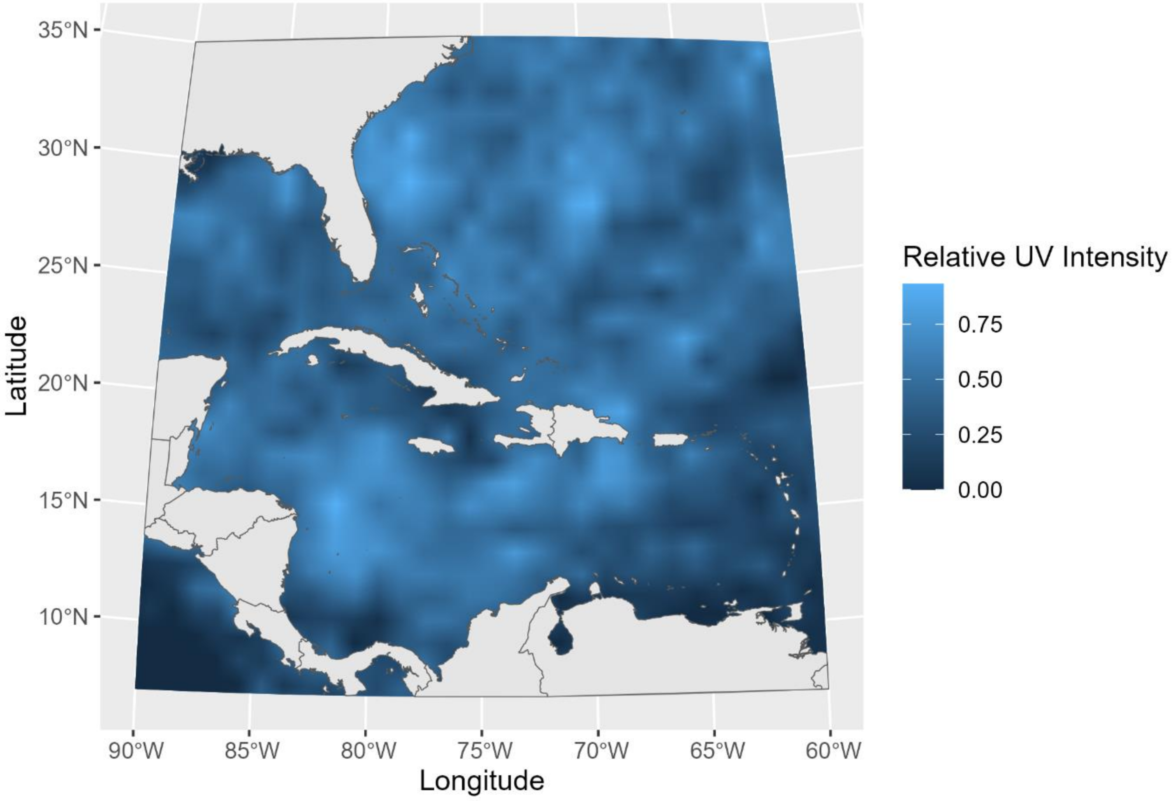

Figure 5. Relative ultraviolet radiation intensity stressor map. Stressor data and methods of collection are available in O'Hara [22]. Dark grey values represent areas where information is not available. Base maps made with Natural Earth.

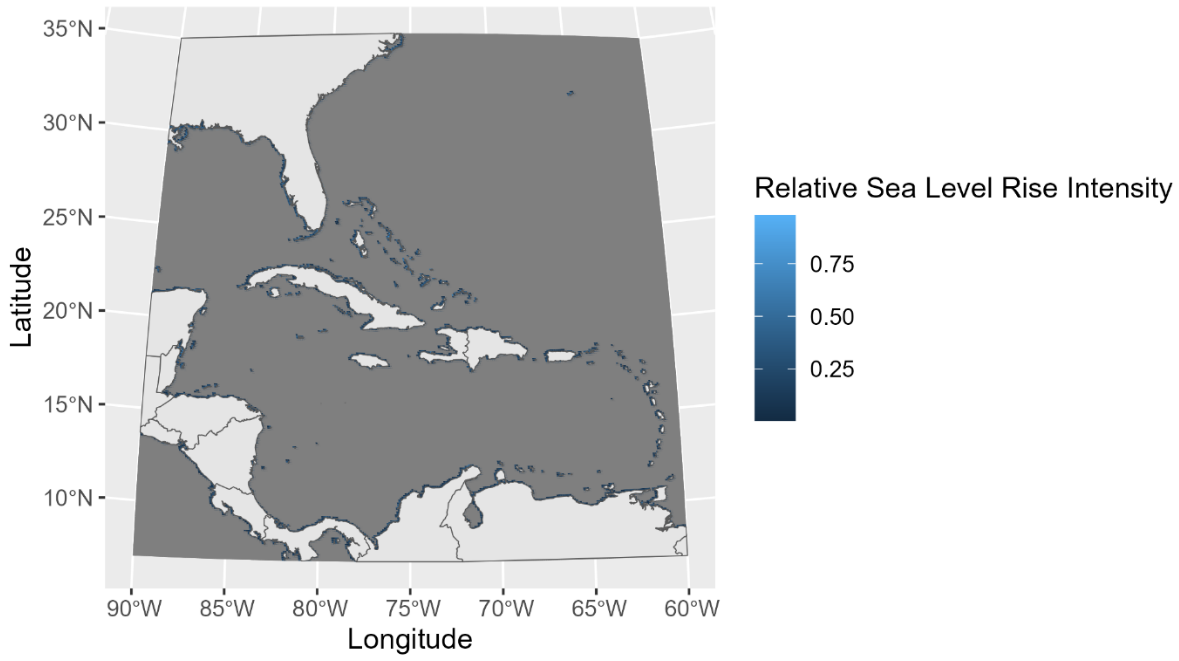

Figure 6. Relative sea level rise intensity stressor map. Stressor data and methods of collection are available in O'Hara [22]. Dark grey values represent areas where information is not available. Base maps made with Natural Earth.

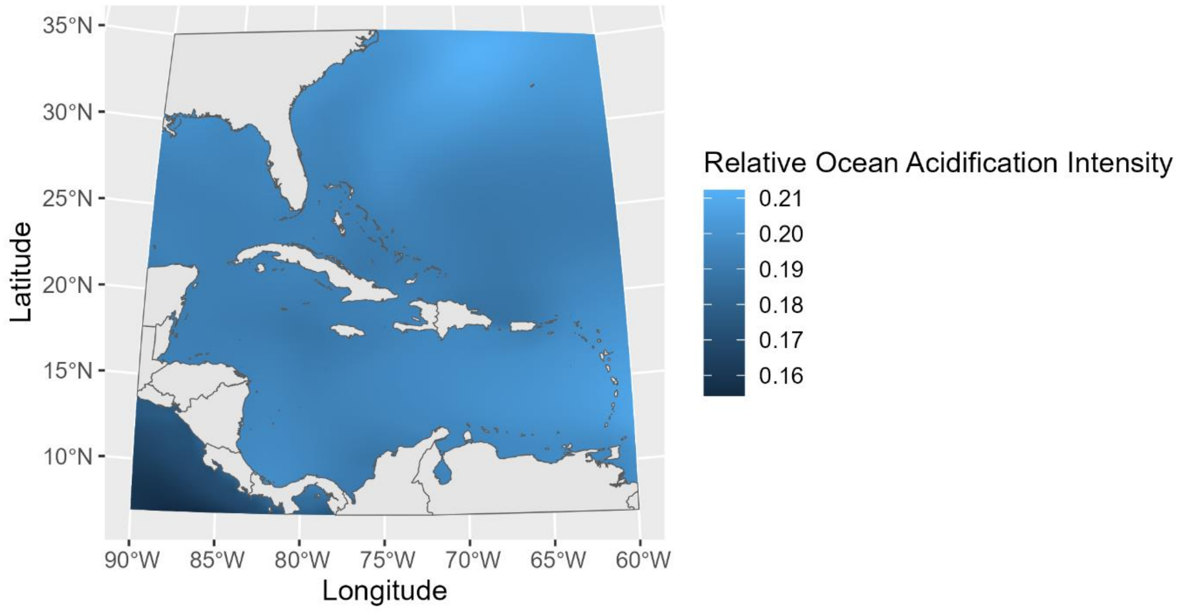

Figure 7. Relative ocean acidification intensity stressor map. Stressor data and methods of collection are available in O'Hara [22]. Dark grey values represent areas where information is not available. Base maps made with Natural Earth.

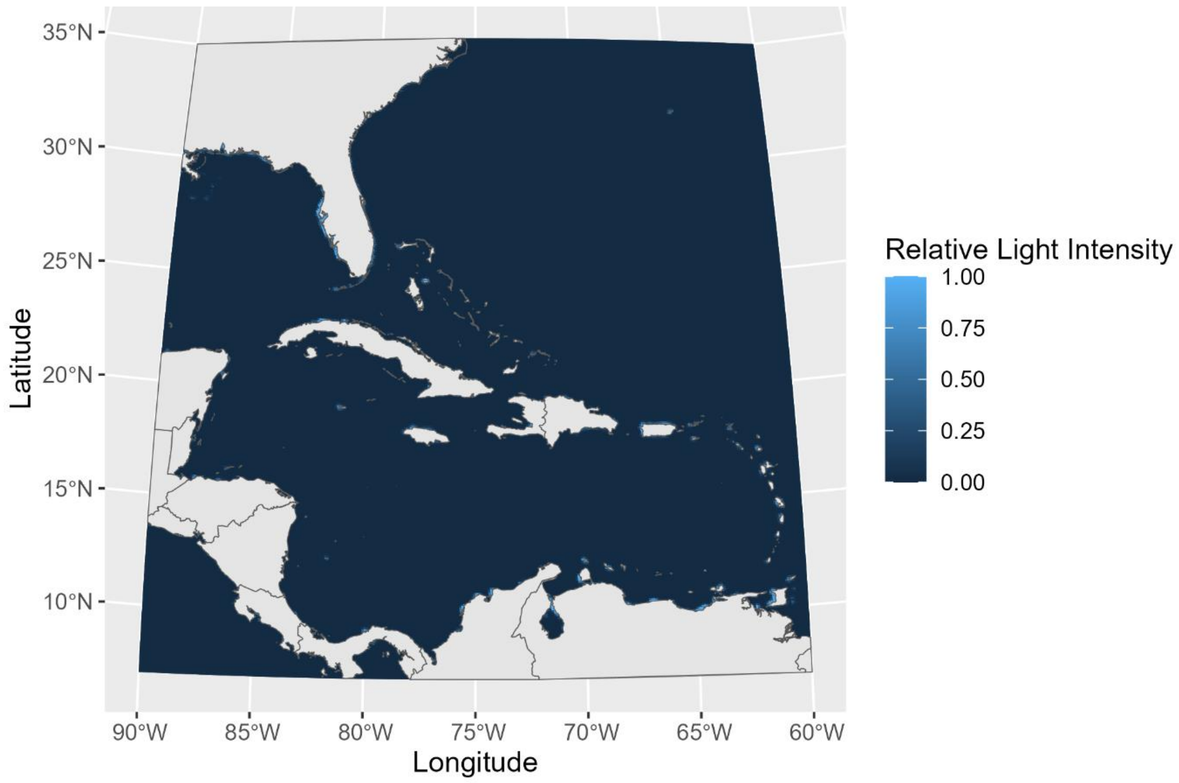

Figure 8. Relative light intensity stressor map. Stressor data and methods of collection are available in O'Hara [22]. Dark grey values represent areas where information is not available. Base maps made with Natural Earth.

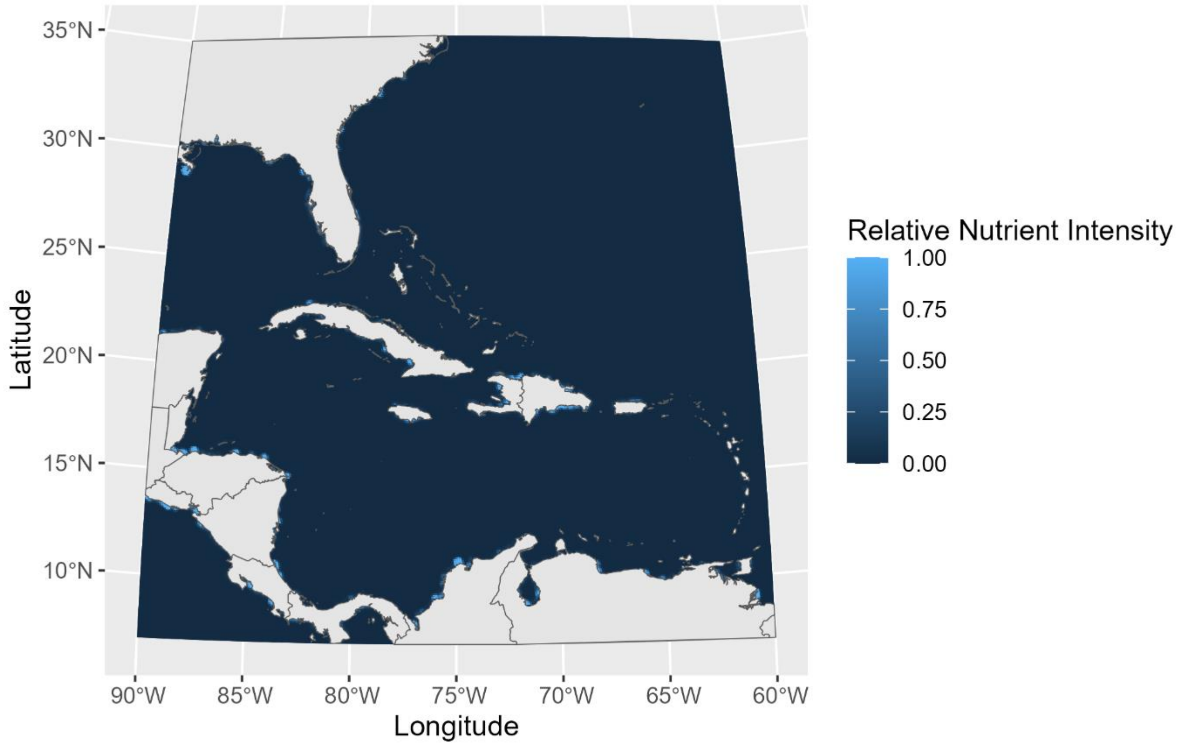

Figure 9. Relative nutrient intensity stressor map. Stressor data and methods of collection are available in O'Hara [22]. Dark grey values represent areas where information is not available. Base maps made with Natural Earth.
