# Supplementary material for: Spatial and life history variation in a trait-based species vulnerability and impact model
Source: PLoS One. 2024 Jun 21;19(6):e0305950. doi: 10.1371/journal.pone.0305950 (PMC11192397; doi:10.1371/journal.pone.0305950)
Supplement: S2 Appendix — (PDF) [file pone.0305950.s003.pdf]

PLOS One

Spatial and life history variation in a trait-based species vulnerability and impact model

Aharon G. Fleury, Casey C. O'Hara, Nathalie Butt, Jaime Restrepo, Benjamin S. Halpern, Carissa J. Klein, Caitlin D. Kuempel, Kaitlyn M. Gaynor, Lily K. Bentley, Anthony J. Richardson, Daniel C. Dunn

S2 Appendix

Boxplots of the impact estimates for seven of the nine stressors. Boxplots for ocean acidification and ultraviolet radiation are not provided as the trait-based vulnerability was zero for every life stage.

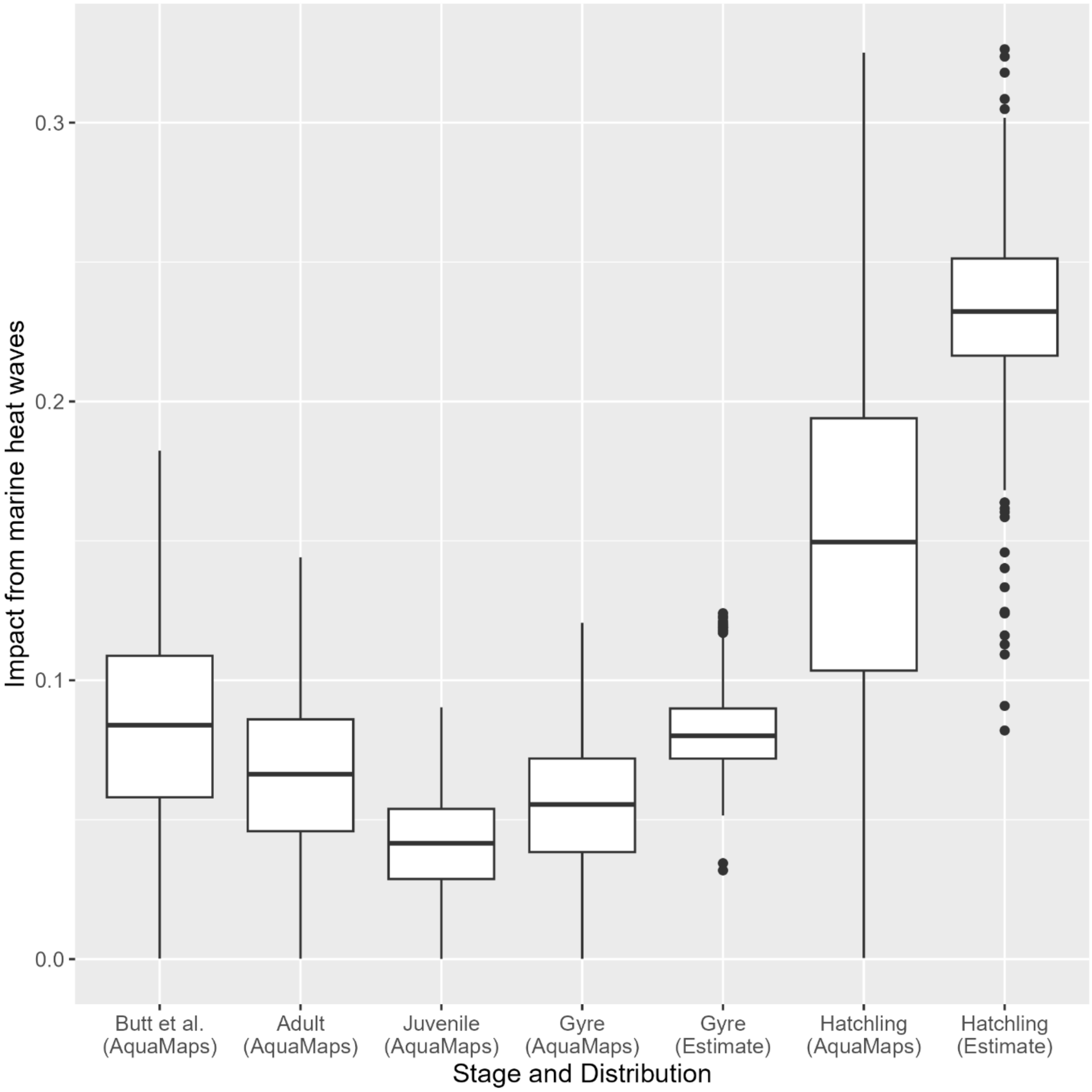

Figure 1. Impact from marine heat waves. AquaMaps distributions represent impact calculations using the AquaMaps distribution. Estimate distributions represent impact calculations using the gyre or hatchling estimate distributions, respectively.

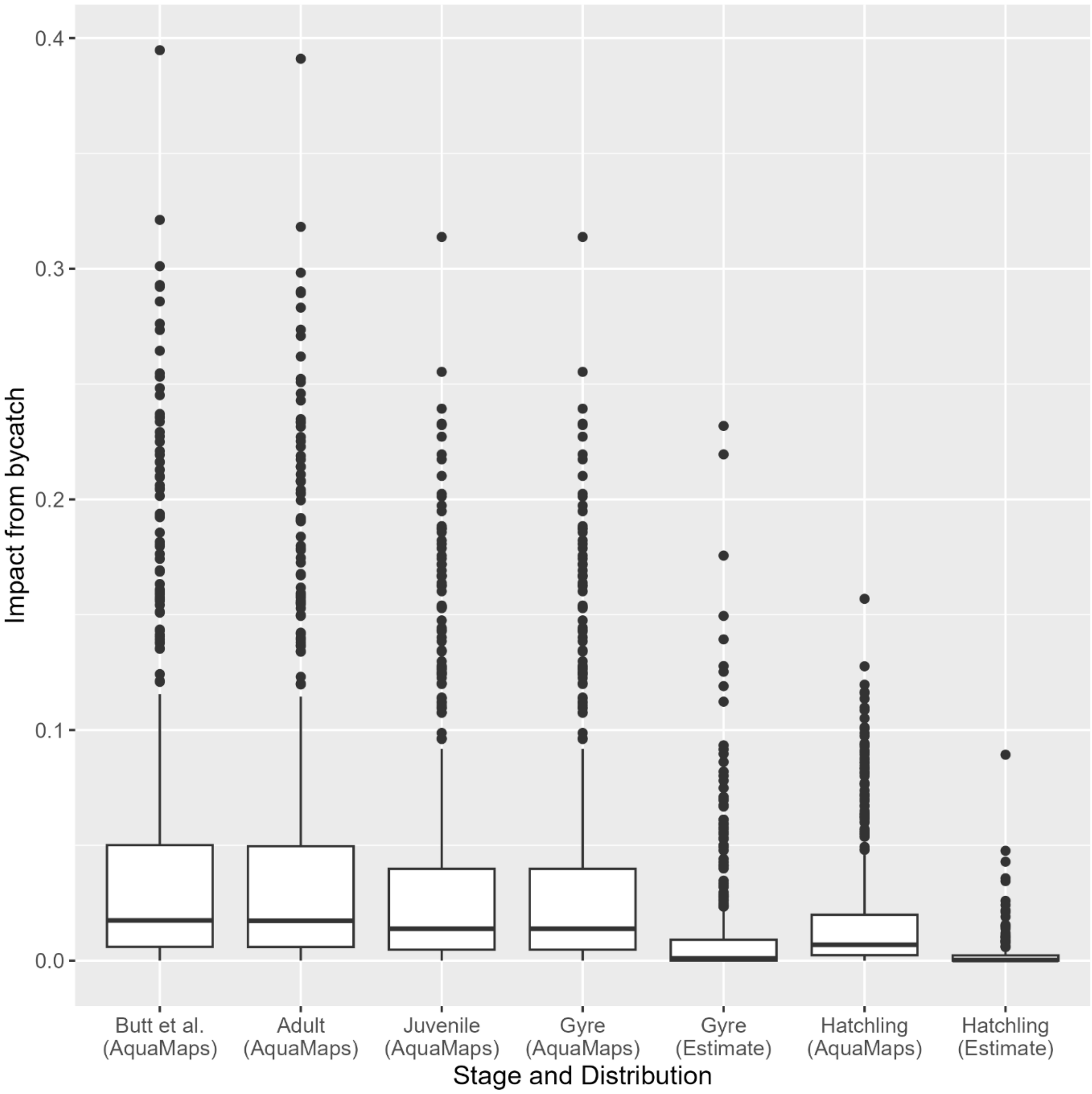

Figure 2. **Impact from benthic and pelagic bycatch.** AquaMaps distributions represent impact calculations using the AquaMaps distribution. Estimate distributions represent impact calculations using the gyre or hatchling estimate distributions, respectively.

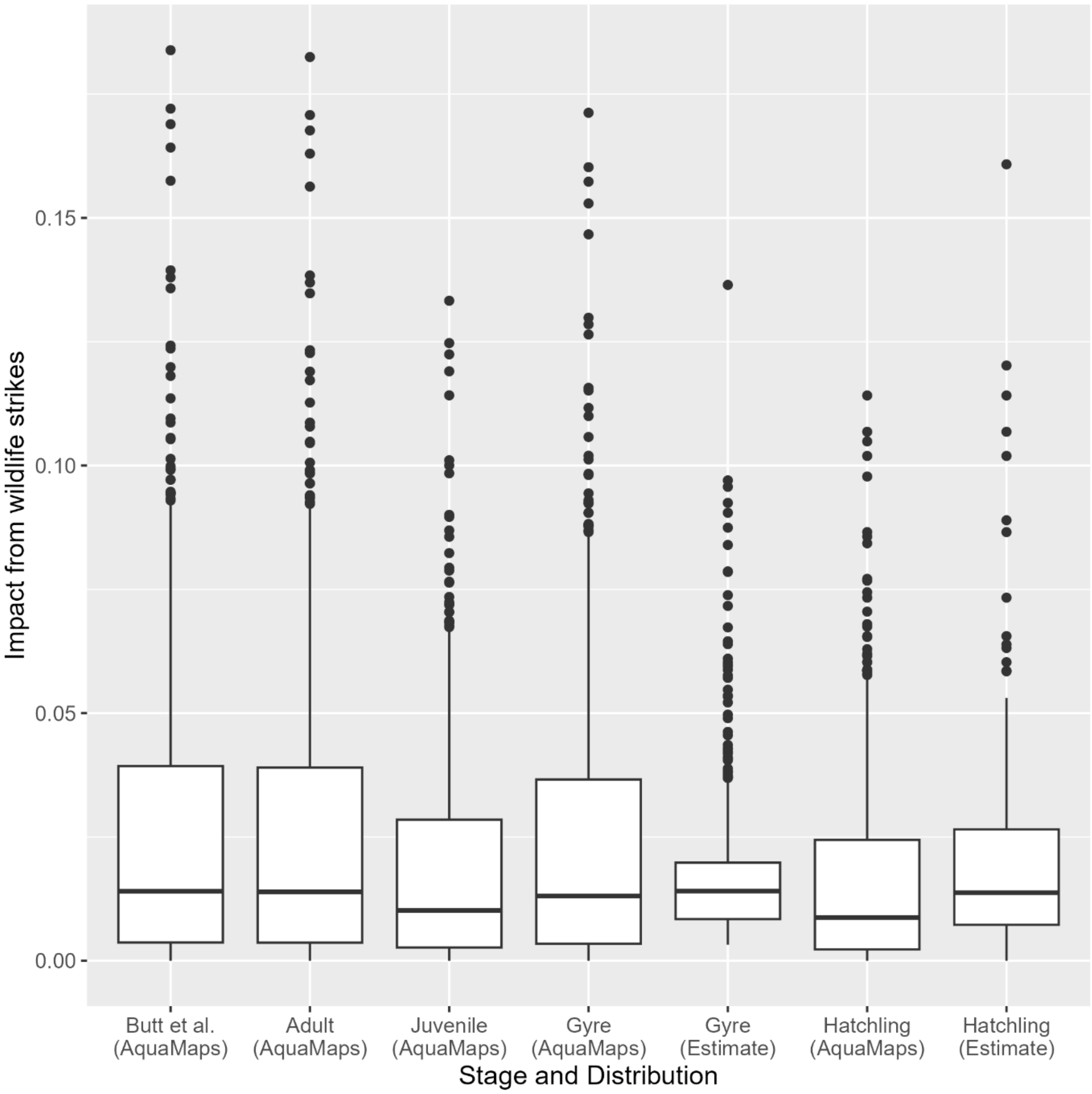

Figure 3. Impact from wildlife strikes. AquaMaps distributions represent impact calculations using the AquaMaps distribution. Estimate distributions represent impact calculations using the gyre or hatchling estimate distributions, respectively.

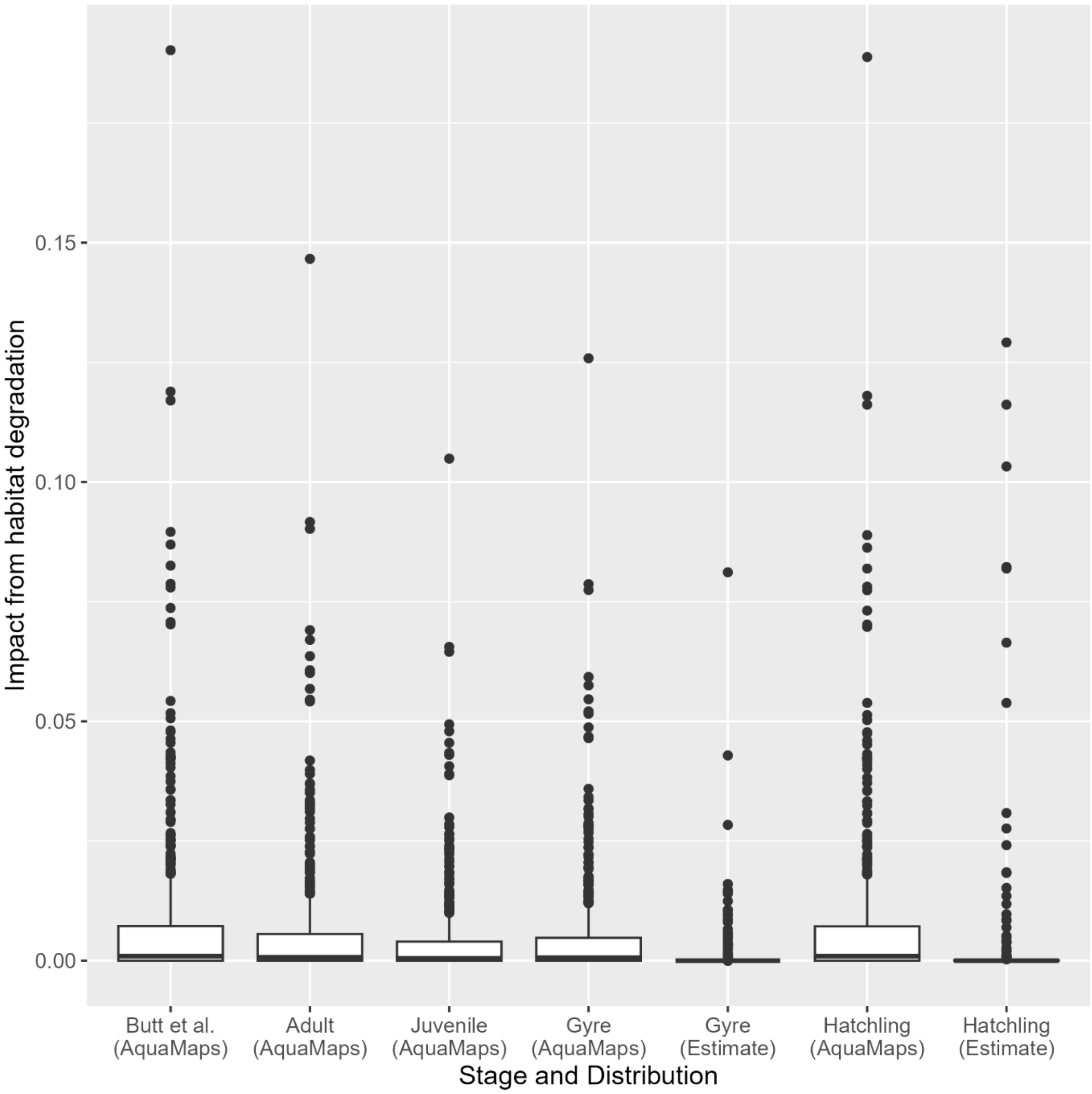

Figure 4. Impact from the habitat degradation. AquaMaps distributions represent impact calculations using the AquaMaps distribution. Estimate distributions represent impact calculations using the gyre or hatchling estimate distributions, respectively.

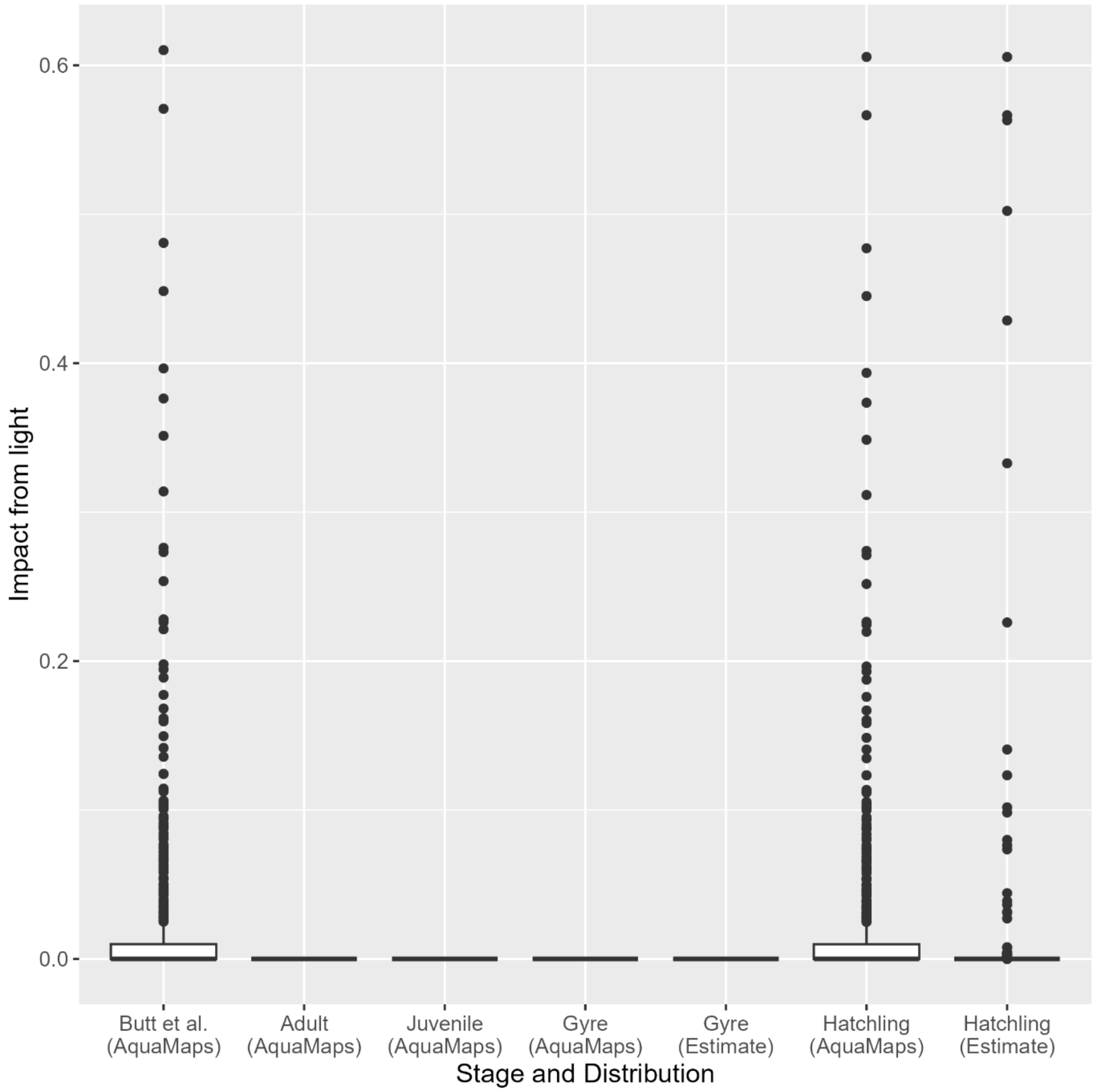

Figure 5. Impact from light. AquaMaps distributions represent impact calculations using the AquaMaps distribution. Estimate distributions represent impact calculations using the gyre or hatchling estimate distributions, respectively.

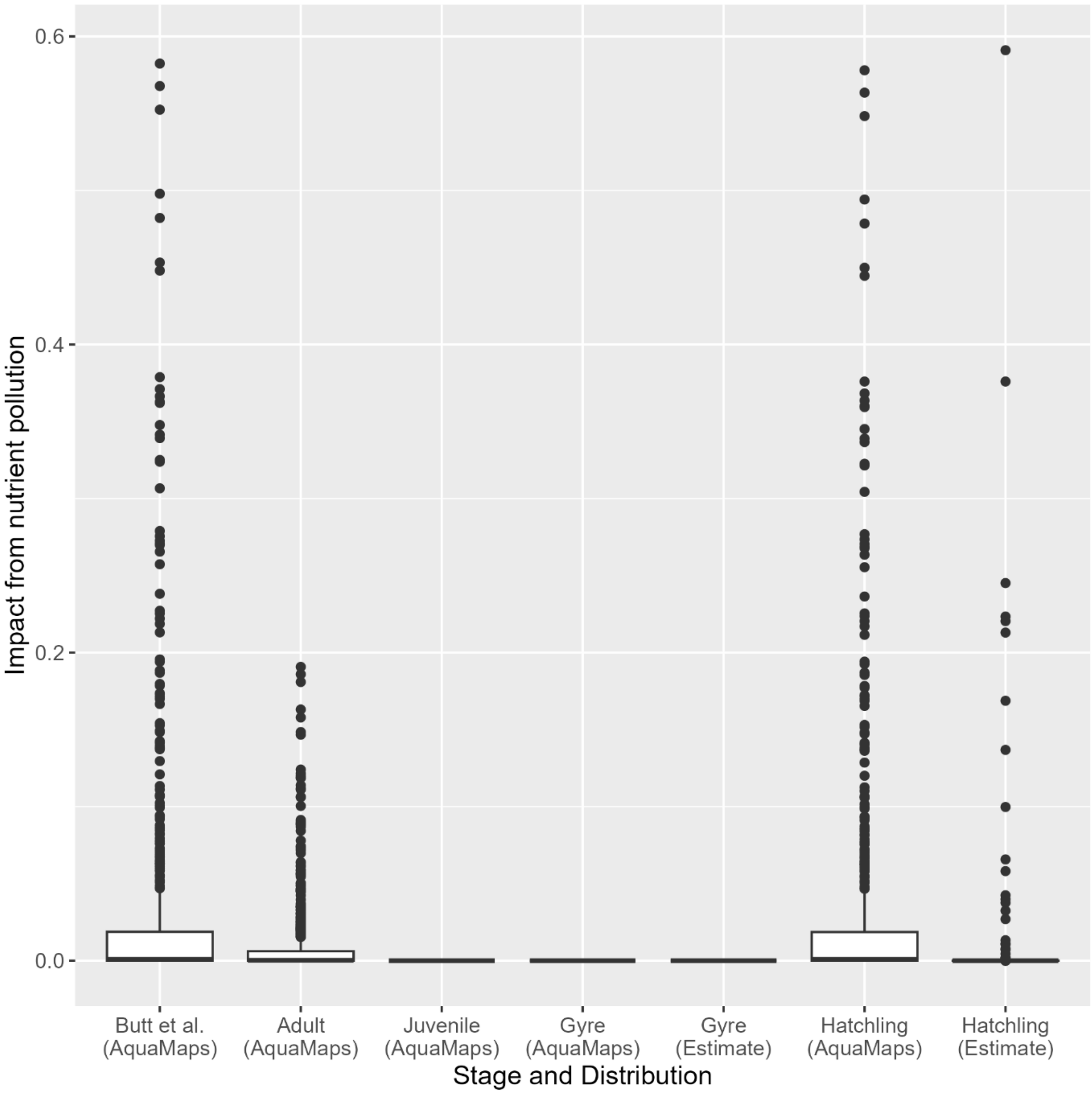

Figure 6. Impact from nutrient pollution. AquaMaps distributions represent impact calculations using the AquaMaps distribution. Estimate distributions represent impact calculations using the gyre or hatchling estimate distributions, respectively.

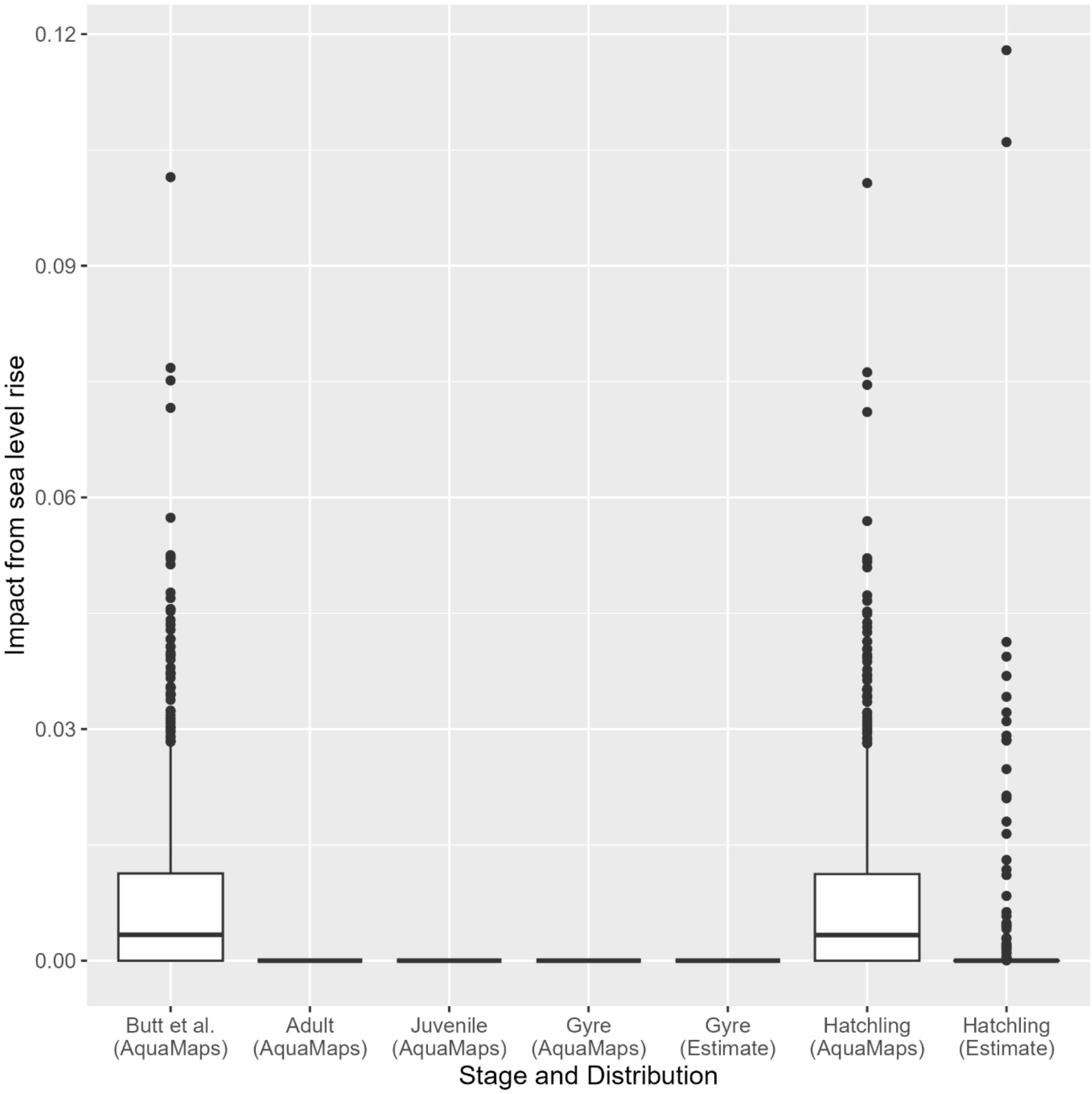

Figure 7. Impact from sea level rise. AquaMaps distributions represent impact calculations using the AquaMaps distribution. Estimate distributions represent impact calculations using the gyre or hatchling estimate distributions, respectively.
